# Green synthesis of fluorescent carbon dots from spices for in vitro imaging and tumour cell growth inhibition

**DOI:** 10.3762/bjnano.9.51

**Published:** 2018-02-13

**Authors:** Nagamalai Vasimalai, Vânia Vilas-Boas, Juan Gallo, María de Fátima Cerqueira, Mario Menéndez-Miranda, José Manuel Costa-Fernández, Lorena Diéguez, Begoña Espiña, María Teresa Fernández-Argüelles

**Affiliations:** 1 Life Sciences Department, International Iberian Nanotechnology Laboratory (INL), Avenida Mestre José Veiga, 4715-330 Braga, Portugal; 2 UCIBIO-REQUIMTE, Laboratory of Toxicology, Biological Sciences Department, Faculty of Pharmacy, University of Porto, Rua de Jorge Viterbo Ferreira, 228, 4050–313 Porto, Portugal; 3 Center of Physics, University of Minho, 4710-057 Braga, Portugal; 4 Department of Physical and Analytical Chemistry, University of Oviedo Julian Clavería 8, 33006 Oviedo, Spain

**Keywords:** bioimaging, carbon quantum dots, fluorescence, spices

## Abstract

Carbon dots have demonstrated great potential as luminescent nanoparticles in bioapplications. Although such nanoparticles appear to exhibit low toxicity compared to other metal luminescent nanomaterials, today we know that the toxicity of carbon dots (C-dots) strongly depends on the protocol of fabrication. In this work, aqueous fluorescent C-dots have been synthesized from cinnamon, red chilli, turmeric and black pepper, by a one-pot green hydrothermal method. The synthesized C-dots were firstly characterized by means of UV–vis, fluorescence, Fourier transform infrared and Raman spectroscopy, dynamic light scattering and transmission electron microscopy. The optical performance showed an outstanding ability for imaging purposes, with quantum yields up to 43.6%. Thus, the cytotoxicity of the above mentioned spice-derived C-dots was evaluated in vitro in human glioblastoma cells (LN-229 cancer cell line) and in human kidney cells (HK-2 non-cancerous cell line). Bioimaging and viability studies were performed with different C-dot concentrations from 0.1 to 2 mg·mL^−1^, exhibiting a higher uptake of C-dots in the cancer cultures compared to the non-cancerous cells. Results showed that the spice-derived C-dots inhibited cell viability dose-dependently after a 24 h incubation period, displaying a higher toxicity in LN-229, than in HK-2 cells. As a control, C-dots synthesized from citric acid did not show any significant toxicity in either cancerous or non-cancerous cells, implying that the tumour cell growth inhibition properties observed in the spice-derived C-dots can be attributed to the starting material employed for their fabrication. These results evidence that functional groups in the surface of the C-dots might be responsible for the selective cytotoxicity, as suggested by the presence of piperine in the surface of black pepper C-dots analysed by ESI-QTOF-MS.

## Introduction

Recent developments in nanotechnology have led to a new generation of high-value optical probes that are being exploited in order to overcome the limitations of traditional dyes and fluorophores. Their great potential has allowed for the development of new analytical assays with unprecedented analytical performance, related to sensitivity, multiplexing capabilities, cost-effectiveness and ease of use [1]. Although inorganic semiconductor quantum dots are the most widely studied fluorescent nanoparticles in bioimaging, biosensing and drug delivery applications, carbon-based ultra-small nanoparticles including carbon quantum dots (C-dots) and graphene quantum dots (GQDs) are emerging as new alternatives due to their excellent properties, including high photoluminescence, low photobleaching, high biocompatibility and low toxicity. C-dots avoid the use of heavy metals present in semiconductor quantum dots, which have raised important health and environmental hazard concerns [2]. Furthermore, due to their ultra-small size, the Brownian motion provides enough energy to prevent nanoparticle aggregation, giving rise to an excellent solubility and stability in aqueous media [3–4]. Their excitation wavelength-dependent emission, their environmental compatibility and water solubility without the need of performing surface chemistry after their synthesis make them the perfect candidates for optical bioimaging and other biomedical applications [5–7].

The fluorescence mechanism of C-dots is not fully understood and there is an ongoing debate on the origin of the emission of C-dots. In fact, it is well known that C-dots synthesized using different synthetic routes, precursors or modifications show different optical performance, which indicates that C-dots are more complex than expected. For instance, the origin of the emission of the C-dots has been attributed to surface state emission, intrinsic band emission, triple ground state emission, dipole emission involving electron–phonon coupling, transition from surface electrons to valence holes, self-trapped excitons and to the presence of small organic molecules. Moreover, the characteristic excitation-dependent emission typically observed in C-dots has been attributed to the presence of multi-emission centres, C-dot size distribution, slow solvent relaxation and the existence of multi-aggregation [8–9]. Also, the optical properties of C-dots are strongly dependent on their local environment, and depending on the surface structure, interactions with the environment can be very selective and reversible. Changes of the optical properties have been attributed to electron transfer from the C-dots to other species, and it has been suggested that the solvent plays an important role due to solvation interactions [10]. Nevertheless, there is still no unanimous agreement in the scientific community about a consistent explanation of the optical properties of C-dots [11].

Nowadays, multiple synthesis techniques are described to obtain C-dots, as well as different carbon sources as alternative for graphite. Generally, the synthesis of C-dots is a multistep and tedious procedure, which is often expensive. Moreover, a surface passivation with other ligands or additives is frequently needed in order to obtain exacerbated intrinsic fluorescence properties [5,12–13]. Recently, green synthesis methods of C-dots based on the use of natural precursors have received much attention since these routes are simple, cost-effective, and the obtained C-dots are highly soluble in water. Recently, among the natural source materials that can be used for the production of C-dots, food products have been studied due to the simple, cost-effective and environmentally friendly hydrothermal process involved in the synthesis [14].

Among the wide variety of food products, common spices like cinnamon, red chilli, turmeric and black pepper have been studied in detail due to their traditionally known medicinal properties. For instance, piperine, a major chemical compound present in black pepper, has shown anti-inflammatory, anti-angiogenic, and anti-arthritic effects [15]. Moreover, it has been reported that black pepper is capable to reduce breast cancer cell proliferation [16–18]. Turmeric is an abundant medicinal herb majorly cultivated in Asia and is widely used in food industries as a colouring agent or food additive [19]. One of its major components, curcumin [20], plays an important role in the treatment of periodontal diseases and oral cancers [21]. Turmeric exhibits numerous therapeutic properties such as antioxidant, anti-inflammatory, anticancer, antiviral and antibacterial activities [22]. Red chilli is another common spice the main pungent ingredient of which is capsaicin. It is used to alleviate neuropathic pain and itching in humans. Moreover, its anticancer properties have been reported in the literature since it has shown to be capable to suppress carcinogenesis of the skin, colon, lung, tongue, and prostate [23–25]. Another spice that has shown promise in preventing and treating cancer is cinnamon [26]. Major constituents in cinnamon include cinnamaldehyde and eugenol [27]. The ability of cinnamon extracts to suppress the growth of gastric cancers has been also reported [28–29].

When talking about the toxicity of C-dots, in vitro and in vivo results reported in the literature are inconsistent. In fact, very recently Pierrat et al. claimed that the toxicity of C-dots is mainly determined by the synthesis protocol [30]. Also, it has been reported that some food-based C-dots show anticancer properties, which strongly relies on the starting material employed for the synthesis [31–32]. Keeping the aforementioned fascinating medicinal activities of selected spices in mind, highly fluorescent C-dots have been synthesized by a green one-pot hydrothermal route, using cinnamon, red chilli, turmeric and black pepper as starting materials. These C-dots based on natural precursors do not exhibit any significant toxicity to non-cancerous cells [27–28], nevertheless it is expected that major compounds present in the spices will partially remain inside or at the surface of the C-dots after the hydrothermal process, leading to different photoluminescent and biomedical properties. The synthesized C-dots have been extensively characterized with UV–vis, fluorescence, FTIR and Raman spectroscopy, DLS, TEM and ESI-QTOF-MS. Moreover, their bioimaging potential and toxicity have been evaluated in vitro in human glioblastoma LN-229 cells and in immortalized epithelial human kidney cells (HK-2). The effects on cancer and non-cancer cells have been also compared with C-dots synthesized from citric acid.

## Results and Discussion

### Absorption and photoluminescence characterization of C-dots

**Figure 1:**
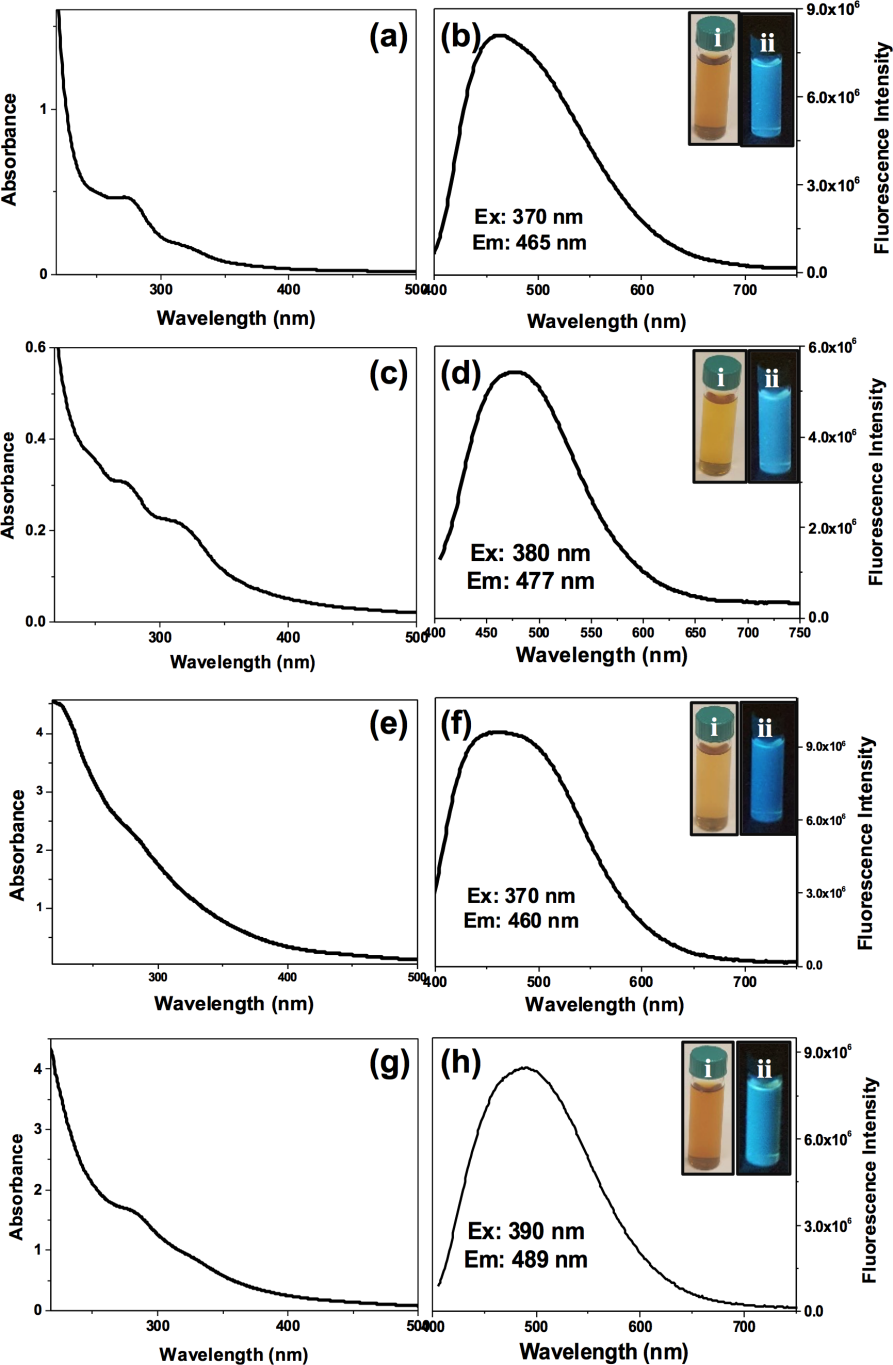
UV–vis absorption and emission spectra of (a, b) cinnamon C-dots, (c, d) red chilli C-dots, (e, f) turmeric C-dots and (g, h) black pepper C-dots (g, h) with their optimum excitation and emission wavelenghts. Insets: Photographs of the corresponding C-dots (i) day light and (ii) UV light.

The C-dots were synthesized using spices as starting material through a green one-pot hydrothermal method that involves pyrolysis, carbonization and passivation [33–35], with no need to add surface passivation agents or any other additives. The obtained C-dots were characterized by UV–vis spectrophotometry and fluorescence spectroscopy. UV–vis spectra of each type of spice-derived C-dots reveal two absorption bands (Figure 1, left column). Cinnamon C-dots show characteristic absorption bands at 275 and 324 nm, red chilli C-dots at 273 and 315 nm, turmeric C-dots at 282 and 329 nm, and black pepper C-dots at 279 and 329 nm. The first absorption band at 270–290 nm is attributed to the π–π* electron transition of C=C bonds (sp^2^ domains). The second absorption band (310–400 nm) corresponds to the n–π* electron transition of C–O bonds (non-bonding oxygen states) [36–40].

Fluorescence spectra exhibit the emission maximum (λ_em_) at 465 nm when using an excitation wavelength (λ_ex_) of 370 nm for cinnamon C-dots (Figure 1, right column). Similarly, fluorescence spectra of red chilli, turmeric and black pepper show maximum emission wavelengths at 477 nm (λ_ex_ = 380 nm), 460 nm (λ_ex_ = 370 nm) and 489 nm (λ_ex_ = 390 nm), respectively. All the obtained C-dots show a brownish-yellow colour under day light, and blue emission under UV light (insets of Figure 1). They are stable in aqueous solution up to six months without any loss of their optical properties.

**Figure 2:**
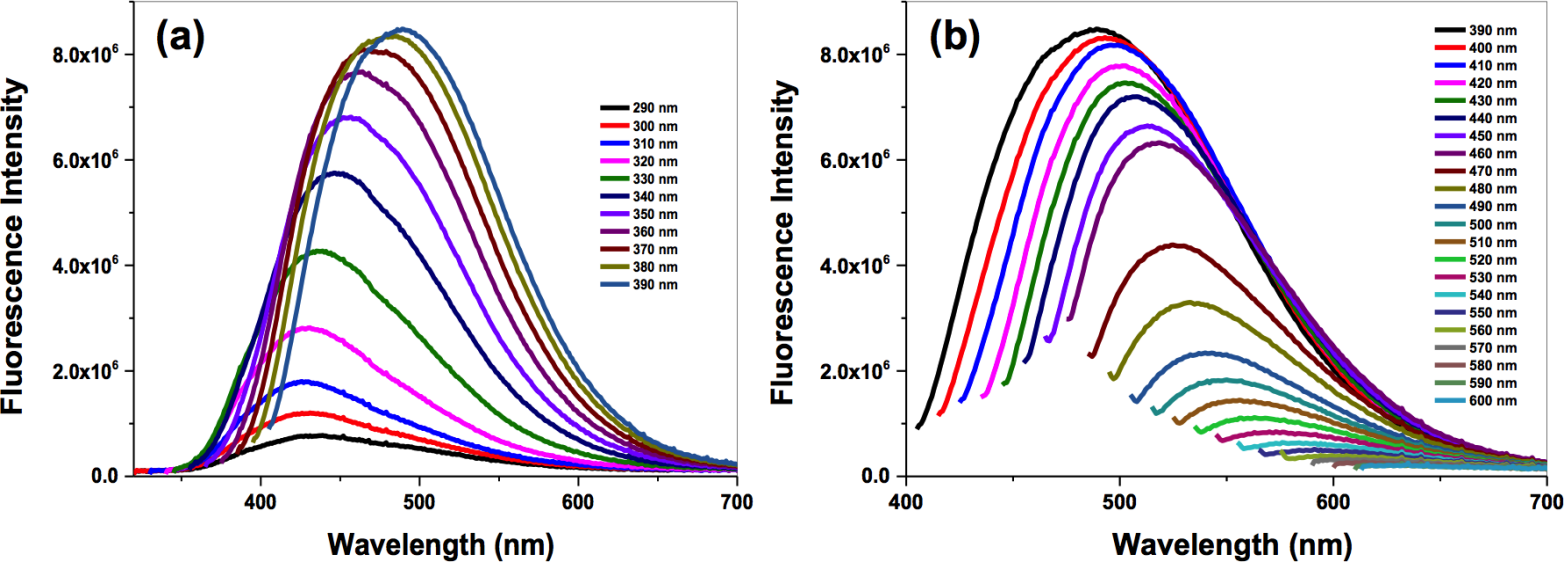
Emission spectra of black pepper C-dots under different excitation wavelengths (a) from 290 to 390 nm and (b) from 390 to 600 nm. Optimum selected conditions are λ_ex_/λ_em_: 390/489 nm.

Typically, C-dots exhibit an interesting excitation-dependent photoluminescence, entirely different from other luminescent materials such as semiconductor quantum dots, gold or silver nanoclusters. This luminescence is attributed to defect states of the C-dots (surface defect emission) and intrinsic defects (zig-zag site emission) [8]. Figure 2 illustrates the emission profile of black pepper C-dots at different excitation wavelengths. The emission spectra were recorded at excitation wavelengths ranging from 290 to 600 nm, and a red shift of the maximum emission wavelength was observed as the excitation wavelength increased. The emission intensity was enhanced as the excitation wavelength increased from 290 to 390 nm (Figure 2a), while excitation wavelengths beyond 390 nm gave rise to a decrease on the fluorescence emission (Figure 2b). The highest fluorescence intensity was observed at 489 nm, when using an excitation wavelength of 390 nm. Hence, 390 nm was selected as the optimum excitation wavelength of black pepper C-dots for further studies. The emission profile of all the synthesized C-dots was also studied, and all of them showed a similar trend (see Figures S1–S3, Supporting Information File 1). Thus, the optimal excitation and emission wavelength combinations selected to perform further experiments were λ_ex_/λ_em_: 370/465 nm for cinnamon C-dots, λ_ex_/λ_em_: 380/477 nm for red chilli C-dots and λ_ex_/λ_em_: 370/460 nm for turmeric C-dots.

Most common sources for the synthesis of carbon dots are graphite and citric acid. Graphite is used in top-down synthesis strategies, and uses dimethylformamide (DMF) as surface passivating agent [8]. However, since DMF is harmful to living cells, in this work carbon dots synthesized from graphite were not selected for control experiments. Also, considering that the C-dots syntheses reported here are based on bottom-up strategies, similar to the synthesis from citric acid, citric acid C-dots have been selected as control in our experiments. It is worth to mention that the citric acid-based C-dots employed in the control experiments have optimum wavelengths of λ_ex_/λ_em_: 380/470 nm. These are very similar (typically of less than 10 nm of spectral shift) to those of the spice-based C-dots described here. The citric acid-based C-dots have been selected because they have been extensively studied in the literature and it has been reported that they are biocompatible.

### TEM, DLS, XRD, FTIR and Raman spectra of C-dots

**Figure 3:**
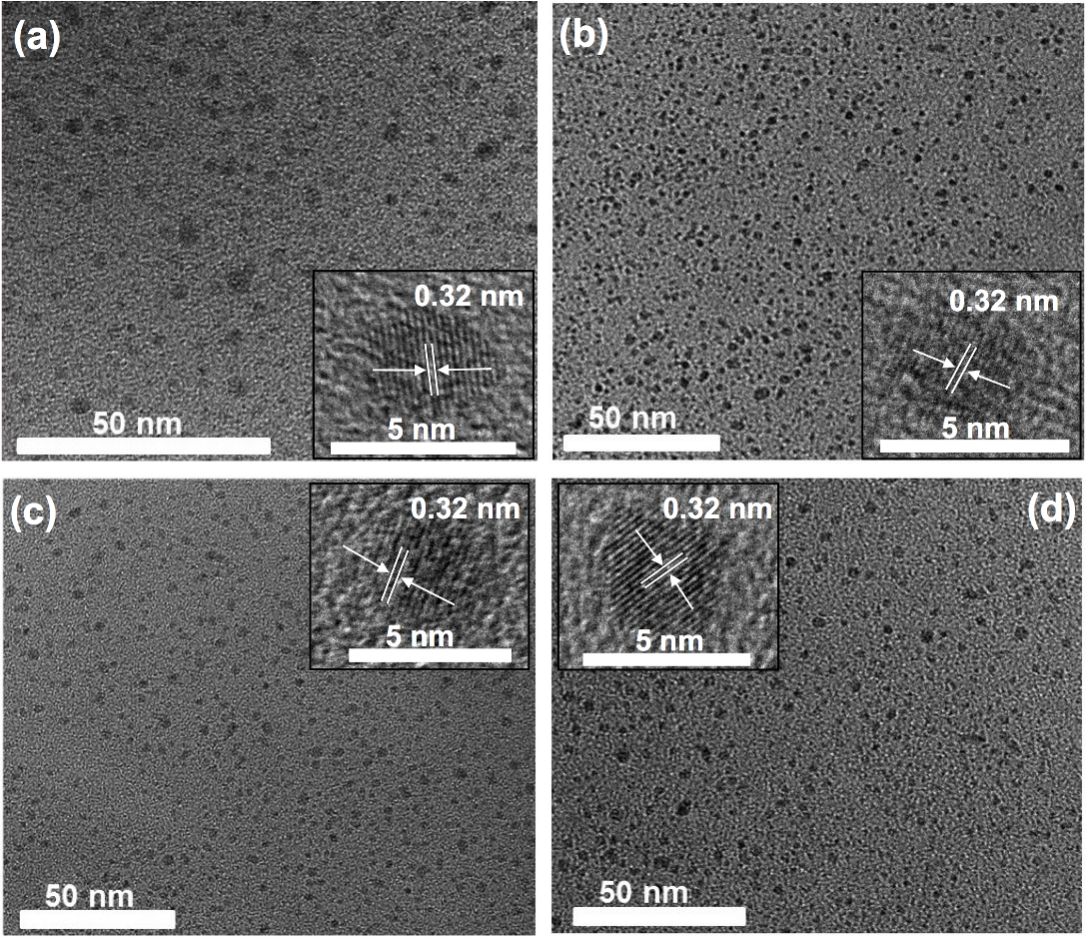
TEM images of (a) cinnamon, (b) red chilli, (c) turmeric and (d) black pepper C-dots. Insets: the crystalline lattices are identified in each corresponding C-dots, and have been estimated as the distance between two parallel dark lines observed in the TEM images.

**Figure 4:**
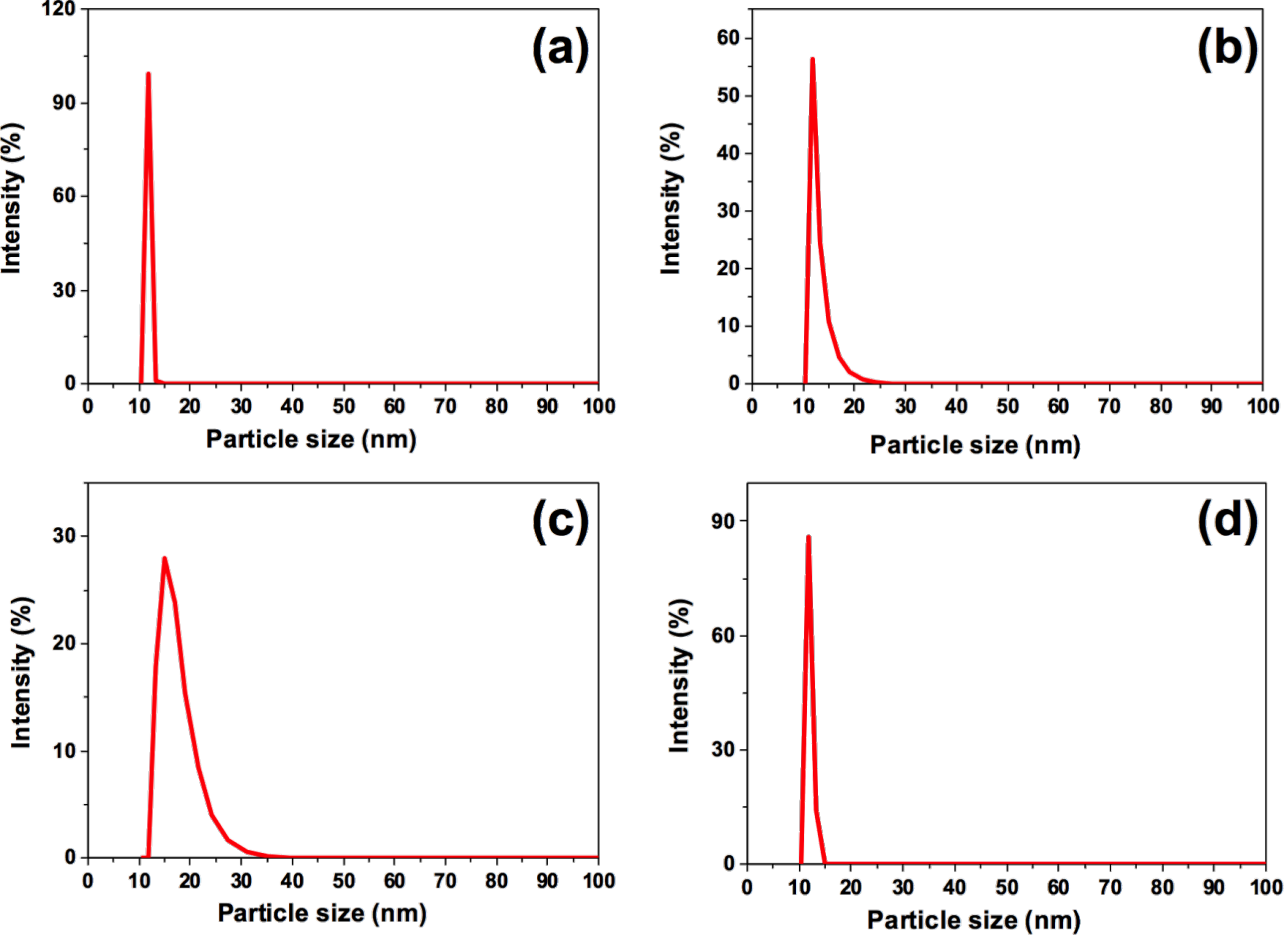
Hydrodynamic radii of (a) cinnamon C-dots, (b) red chilli C-dots, (c) turmeric C-dots and (d) black pepper C-dots obtained through DLS measurements.

TEM images showed that the obtained C-dots are spherical regardless of the starting material. As it can be observed in Figure 3, C-dots are uniform in size and shape. A TEM histogram was plotted to estimate the average diameter of the C-dots, giving rise to 3.4 ± 0.5, 3.1 ± 0.2, 4.3 ± 0.5 and 3.5 ± 0.1 nm for cinnamon, red chilli, turmeric and black pepper C-dots, respectively. Moreover, the C-dots were also characterized by HR-TEM, obtaining a lattice *d*-spacing of 0.32 nm for all the C-dots (inset of Figure 3), which confirms that the obtained C-dots are of crystalline graphitic nature [29–31]. Hydrodynamic radii measured by DLS gave rise to values of 11.0, 10.3, 15.0 and 11.2 nm (Figure 4), and zeta potential values of −16.0, −32.9, −16.3 and −24.2 mV for cinnamon, red chilli, turmeric and black pepper C-dots, respectively. All the synthesized spice-based carbon dots present negative zeta potentials with high absolute values. Such negative values ensure a good colloidal stability of these carbon dots in biological media. However, it must be noticed that there is no trend observed between the absolute value of the zeta potential and the cellular uptake observed in both LN-229 and HK-2 cells.

**Figure 5:**
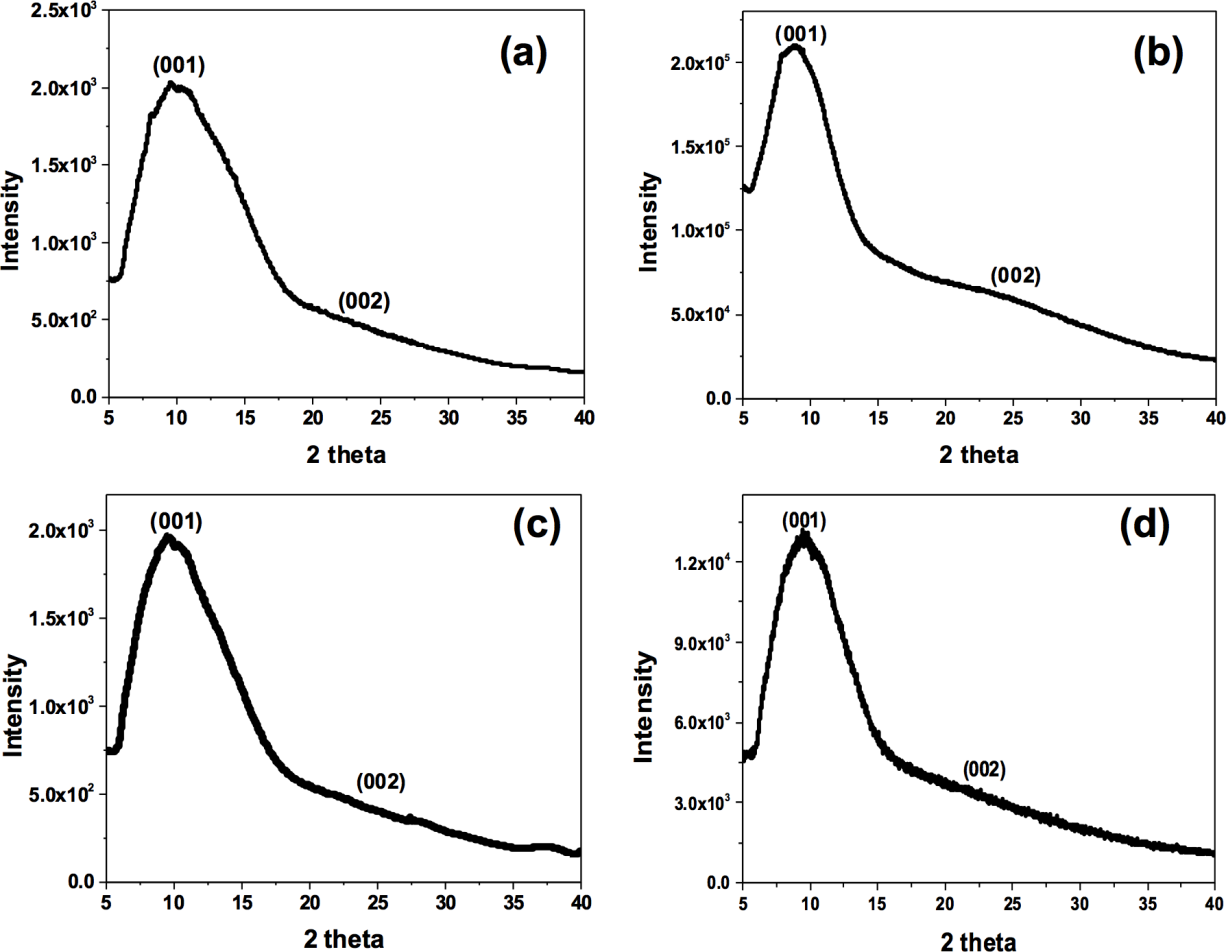
XRD pattern of (a) cinnamon C-dots, (b) red chilli C-dots, (c) turmeric C-dots and (d) black pepper C-dots.

XRD patterns of the four synthesized C-dots have been also studied, and the obtained results are given in Figure 5. The cinnamon, red chilli, turmeric and black pepper C-dots diffraction peaks are located at 9.7, 9.4, 9.0 and 9.8°, respectively, corresponding to the graphitic carbon(001) plane. A broad reflection observed around 25°, which corresponds to the graphitic carbon(002) plane, is due to the small size of the C-dots [41]. These diffraction peaks match well with the characteristic peaks of graphene oxide [42–44], and they are also in agreement with the HR-TEM lattice distances measured.

**Figure 6:**
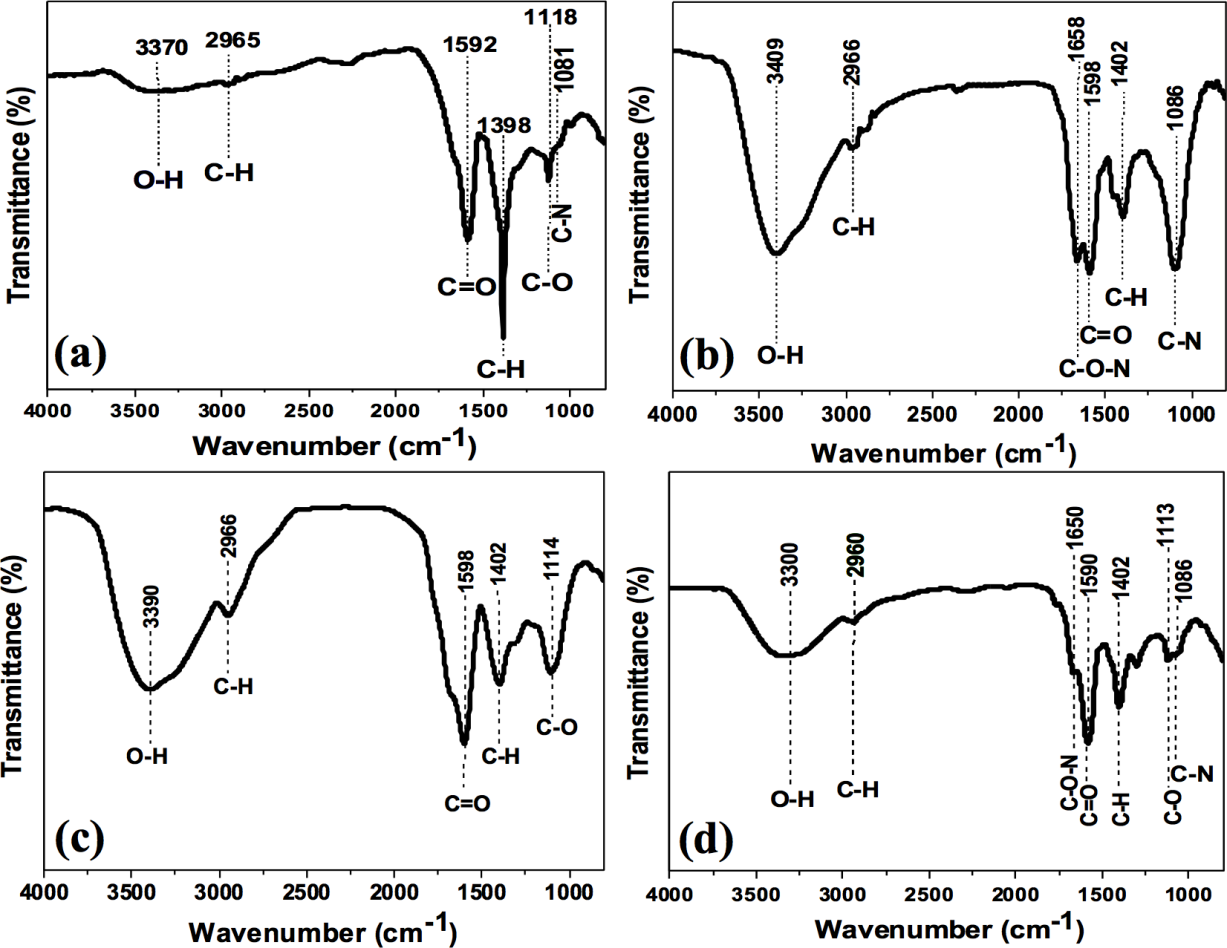
FTIR spectra of (a) cinnamon C-dots, (b) red chilli C-dots, (c) turmeric C-dots and (d) black pepper C-dots.

FTIR spectroscopy of the synthesized C-dots confirms the presence of different functional groups in each sample depending on the starting material. The FTIR spectrum of cinnamon C-dots shows the O–H vibrational stretching and C–H bending peaks at 3370 and 2965 cm^−1^, respectively. C=O, C–H, C–O and C–N vibrational stretching peaks are observed at 1592, 1398, 1118 and 1081 cm^−1^, respectively (Figure 6a) [45]. Figure 6b shows the FTIR spectrum of red chilli C-dots. The peaks observed at 3409 and 2966 cm^−1^ are assigned to O–H vibrational stretching and C–H bending peaks, respectively. C–O–N, C=O, C–H and C–N vibrational stretching peaks are observed at 1658, 1598, 1402 and 1086 cm^−1^, respectively [46]. The FTIR spectrum of turmeric C-dots (Figure 6c) shows peaks at 3390, 2966 and 1598 cm^−1^, which were assigned to the O–H, C–H and C=O vibrational stretching peaks, as well as peaks at 1402 and 114 cm^−1^, which were attributed to C–H and C–O bending peaks, respectively [47]. Finally, the FTIR spectrum of black pepper C-dots (Figure 6d) shows O–H and C–H vibrational stretching peaks around 3300 and 2960 cm^−1^, respectively. The peaks at 1650 and 1590 cm^−1^ were assigned to vibrational stretching peaks of C–O–N and C=O, respectively, and the peaks observed at 1402, 1113 and 1086 cm^−1^ were attributed to C–H, C–O and C–N vibrational stretching peaks [48].

**Figure 7:**
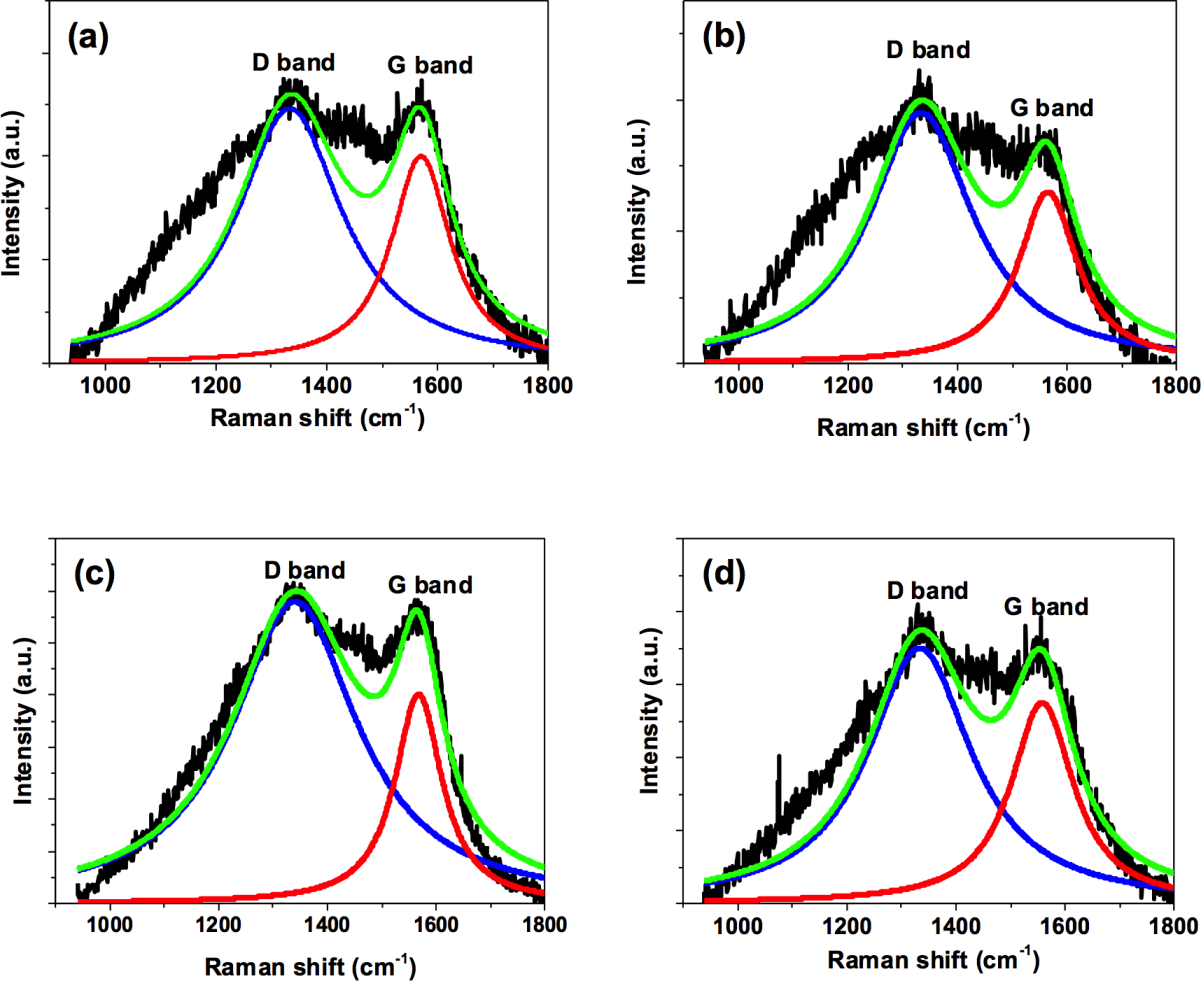
Raman spectra of (a) cinnamon C-dots, (b) red chilli C-dots, (c) turmeric C-dots and (d) black pepper C-dots.

Raman spectra of the spice C-dots were fitted using a Gaussian function, as shown in Figure 7. Cinnamon C-dots present a D band at 1336.5 cm^−1^ and a G band at 1569.1 cm^−1^ (Figure 7a). Red chilli C-dots show the D band at 1338 cm^−1^ and the G band at 1562.5 cm^−1^ (Figure 7b). Turmeric C-dots show the D band at 1340.6 cm^−1^ and the G band at 1567.5 cm^−1^ (Figure 7c), and finally, black Pepper C-dots exhibit the D band at 1339.5 cm^−1^ and the G band at 1554.7 cm^−1^ (Figure 7d). The obtained D band (sp^3^) and G band (sp^2^) correspond to the A_1g_ symmetry photons near the K-zone boundary and E_2g_ the vibrational mode of sp^2^ carbon, respectively. The relative intensities of D band and G band (*I*_D_/*I*_G_) for cinnamon, red chilli, turmeric and black pepper C-dots were 1.1, 1.2, 1.4 and 1.1, respectively, and reveal the existence of vacant lattice sites of sp^3^ carbon [27,49–50].

### Quantum yield measurements

The fluorescent quantum yield of each type of spice C-dots was calculated by using the comparative William’s method [51]. For this purpose, quinine sulphate was employed as a reference and the quantum yield was calculated according to Equation 1:

[1]
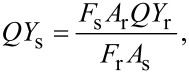


where, *F*_s_ is the integrated fluorescence emission of the sample, *F*_r_ is the integrated fluorescence emission of the reference (quinine sulfate), *A*_s_ is the absorbance at the excitation wavelength of the sample, *A*_r_ is the absorbance at the excitation wavelength of the reference, *QY*_s_ is the quantum yield of the sample, and *QY*_r_ is the quantum yield of the reference fluorophore (quinine sulfate *QY* = 54%). The calculated fluorescence quantum yields of cinnamon, red chilli, turmeric and black pepper C-dots are 35.7, 26.8, 38.3 and 43.6%, respectively. The obtained high quantum yield values confirm that the synthesized C-dots are highly fluorescent. Black pepper C-dots show the highest quantum yield among all of the spice-derived C-dots. A summary of the characteristic parameters studied for each spice C-dot is collected in Table 1.

**Table 1:** Summary of parameters measured for characterization of the synthesized C-dots.

parameter	cinnamon C-dots	red chilli C-dots	turmeric C-dots	black pepper C-dots

λ_ex_//λ_em_ (nm)	370/465	380/477	370/460	390/489
FWHM (nm)	136	125	145	133
Stokes shift (nm)	95	97	90	99
quantum yield	35.7%	26.8%	38.3%	43.6%
Raman (*I*_D_/*I*_G_)	1.1	1.2	1.4	1.1
TEM size (nm)	3.4 ± 0.5	3.1 ± 0.2	4.3 ± 0.5	3.5 ± 0.1
DLS (nm)	11.0	10.3	15.0	11.2
zeta potential (mV)	−16.0	−32.9	−16.3	−24.2

### Cell viability measurements and cell imaging using the C-dots

Concentrations varying from 0.1 mg·mL^−1^ to 2 mg·mL^−1^ (and up to 4 mg·mL^−1^ in the case of black pepper C-dots) were tested in vitro for cytotoxicity in epithelial human kidney cells (HK-2) and in glioblastoma LN-229 cells (results obtained for each type of C-dots are displayed in detail in Figures S5–S9, Supporting Information File 1). Please, notice that the range of carbon dot concentrations used during the in vitro cell viability studies match very well with those assayed in previous works using other types of carbon dots [27].

The highest tested C-dot concentrations correspond to approximately 15% of water; in all cases, cell death due to the water vehicle was excluded by testing cell viability for the highest amount of water (15%) used in the experiments (data not shown). Although the C-dots emission curve at 560 nm excitation wavelength (Figure 2) suggested that these would not interfere with the viability assay, this was further tested by incubating growing concentrations of each C-dot type with cell culture medium containing PrestoBlue (PB) reduced by untreated cells. As shown in Figure S4 (Supporting Information File 1), reading fluorescence emission at 590 nm before and after the addition of C-dots shows no interference of the C-dots on PB emission at 560 nm excitation wavelength.

**Figure 8:**
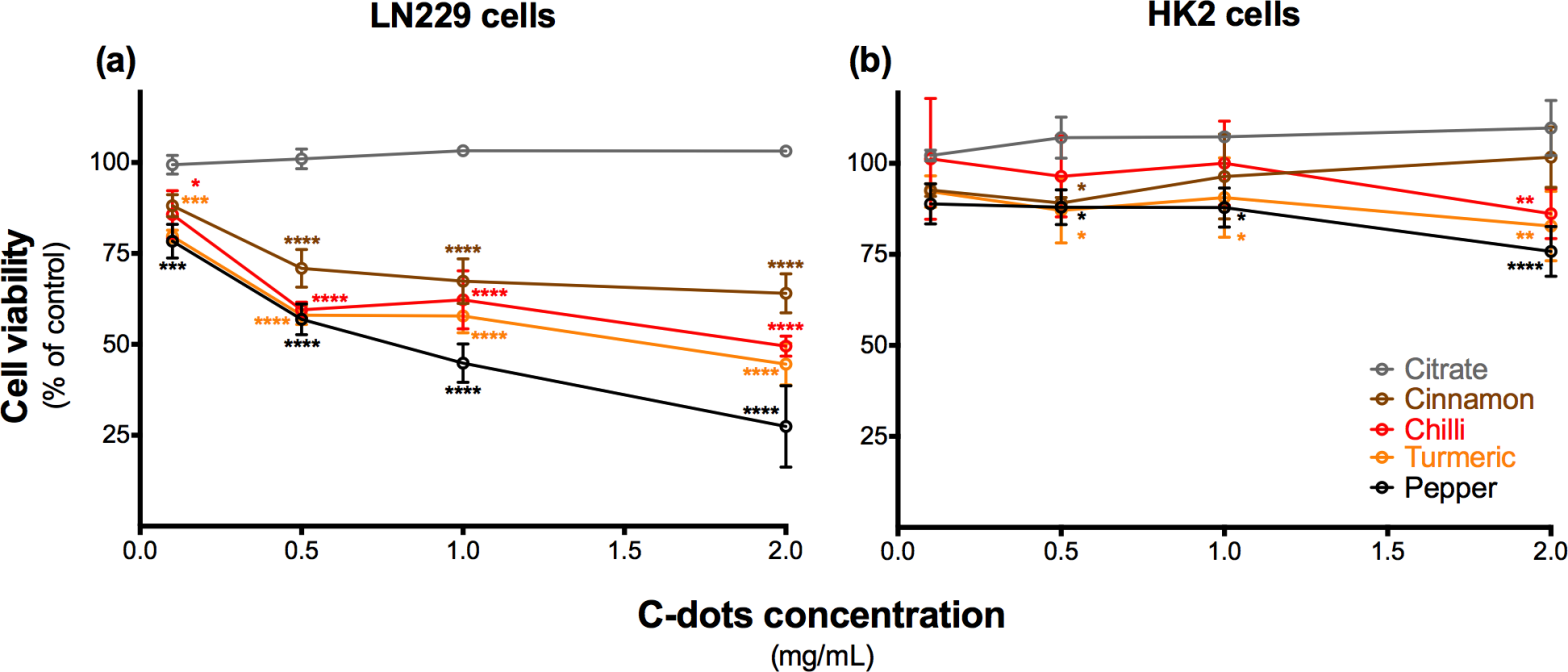
Cell viability evaluation (PrestoBlue) after 24 h of incubation with increasing concentrations of each type of C-dots. After exposing both LN-229 and HK-2 cells, the ability of these cells to metabolize resazurin to resofurin was tested as a measure of cell viability. Results are means ± SD of at least three independent experiments. *p < 0.05, **p < 0.01, ***p < 0.001 and ****p < 0.0001 for statistical significance of differences between the same concentration of citric acid-derived and each of the food-derived C-dots.

A significant decrease in viability of the LN-229 cells was observed after 24 h of exposure to the spice-derived C-dots, when compared to the citric acid (citrate)-derived C-dots used as control (synthesized as described elsewhere [52]), which did not show any effect (Figure 8a). When comparing the effect of the same concentration of C-dots on LN-229 cells, the cinnamon C-dots were the least toxic. For instance, at a dose of 2 mg·mL^−1^ cinnamon C-dots induced around 35% reduction in cell viability, followed by both red chilli and turmeric C-dots, which induced nearly 50% reduction in cell viability for the same concentration. The black pepper C-dots were the most toxic ones, yielding a 75% reduction in cell viability at 2 mg·mL^−1^, and achieving almost 100% cell death of LN-229 cells for the highest concentration tested (4 mg·mL^−1^, Figure S8, Supporting Information File 1). Although the mechanism ruling the cytotoxicity effect exerted by these C-dots was not studied, it has been reported an association between the toxic effects of ginger C-dots and increased ROS production [27], which could also be responsible for the cell toxicity effect in this case.

Interestingly, when testing the same concentrations of spice C-dots in HK-2 cells, a human cell line of non-cancerous renal cells, the effects on cell viability were clearly less pronounced and significantly different from the ones observed for LN-229 cells, in particular for concentrations above 0.5 mg·mL^−1^ (Figure 8b). No more than 15% reduction in HK-2 cell viability was observed, with the exception of black pepper C-dots, which at the higher concentrations were found to be also significantly toxic to this cell line (Figures S5–S9, Supporting Information File 1). Therefore, the susceptibility of HK-2 cell line was found to be significantly different from LN-229 cells for all the spice-derived C-dots, results that are in agreement with those described by other researchers on other types of non-cancerous and tumour cell lines using ginger and green tea-based C-dots [27–28]. The fact that the citrate-derived C-dots did not induce any significant effect on cell viability neither in LN-229 nor in HK-2 cells (Figure 8) suggests that the inhibition effect on the cell growth of LN-229 cells can be attributed to the nature of the spice-based C-dots, indeed depending on the starting material employed for the C-dots synthesis.

**Figure 9:**
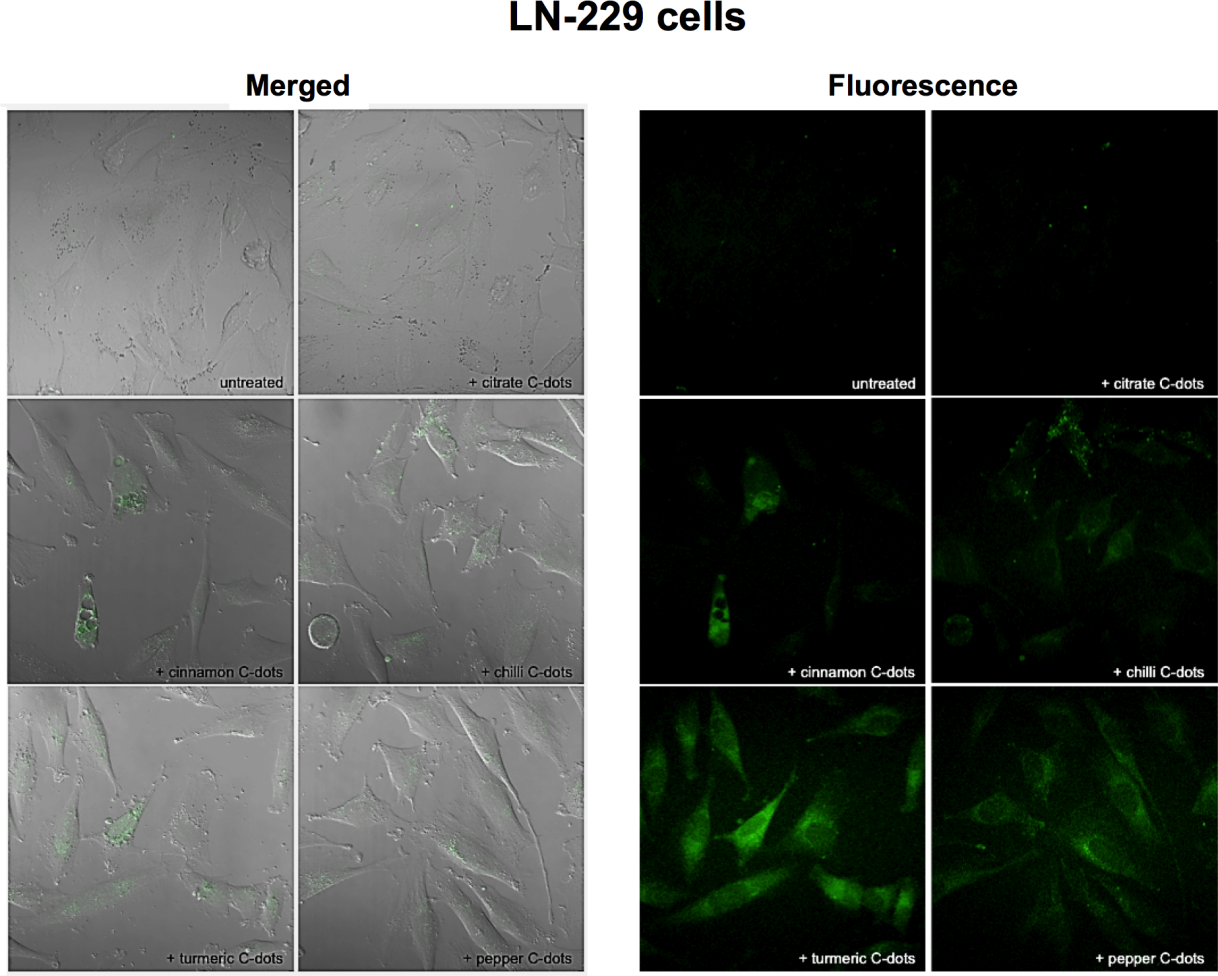
Merged transmission and fluorescence (left) and fluorescence (right) imaging of LN-229 cancer cells after 24 h of incubation with no C-dots (untreated) and with 1 mg·mL^−1^ of citrate, cinnamon, red chilli, turmeric and black pepper C-dots. Images were collected using a Zeiss LSM780 confocal microscope (40× objective), using a 405 nm laser and collecting the emitted fluorescence in the green area.

**Figure 10:**
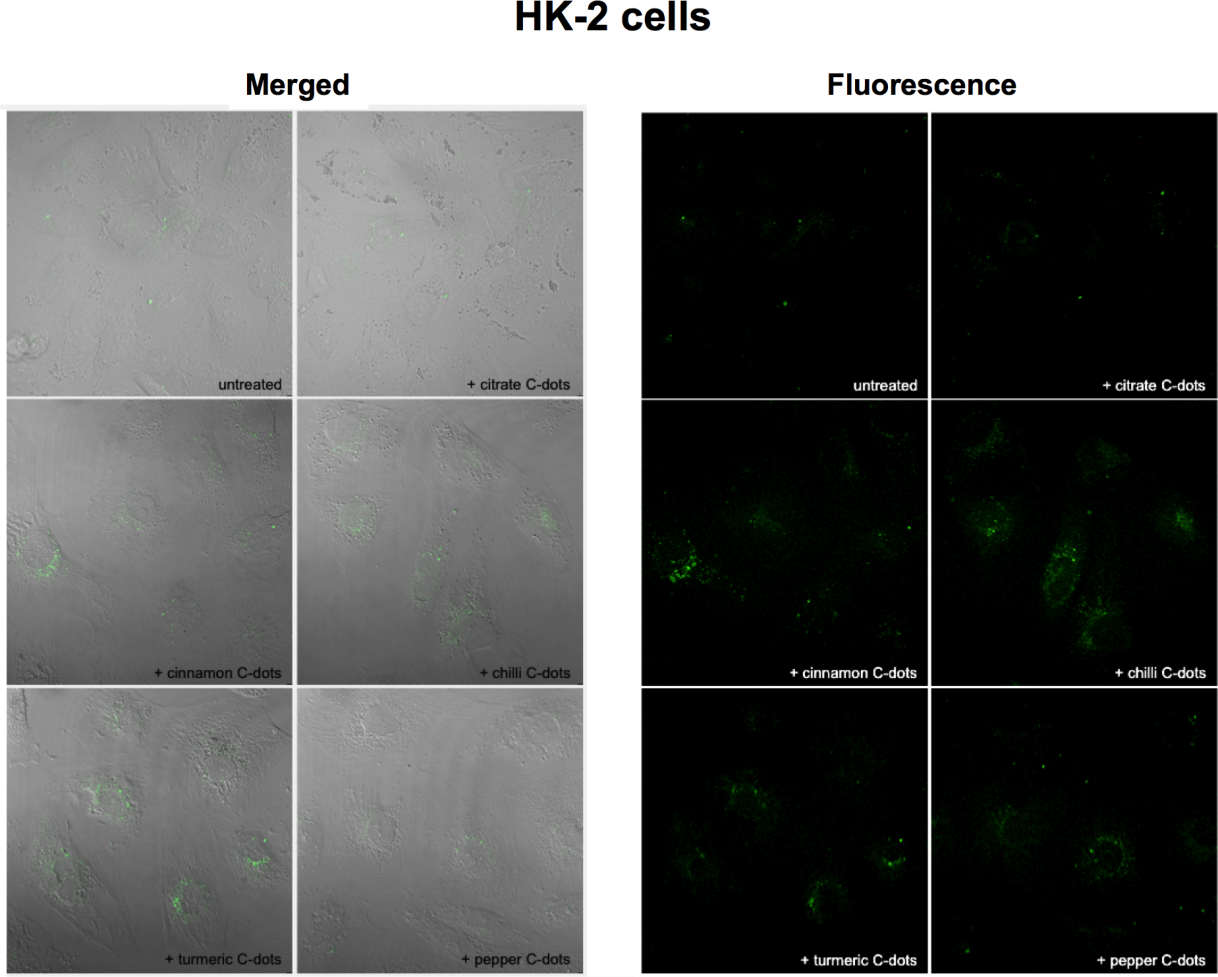
Merged transmission and fluorescence (left) and fluorescence (right) imaging of HK-2 cells after 24 h of incubation with no C-dots (untreated) and with 1 mg·mL^−1^ of citrate, cinnamon, red chilli, turmeric and black pepper C-dots. Images were collected using a Zeiss LSM780 confocal microscope (40× objective), using a 405 nm laser and collecting the emitted fluorescence in the green area.

Considering the results obtained in cell viability studies, fluorescence imaging experiments were conducted after the incubation of LN-229 and HK-2 cells for 24 h, with each C-dot solution (spice- and citrate-based C-dots) at a concentration of 1 mg·mL^−1^. Confocal fluorescence imaging shows a diffuse accumulation of the C-dots in the cytoplasm of LN-229 cells (Figure 9), while distributed in bigger agglomerates around the nuclear area in HK-2 cells (Figure 10). The fluorescence intensity attributed to the C-dots was clearly higher in LN-229 cells than in HK-2 cells, suggesting a more efficient uptake by LN-229 than by HK-2 cells, which may also contribute to the observed different susceptibilities. Moreover, as can be seen in the top left images of Figure 9 and Figure 10, a negligible autofluorescence was observed in untreated HK-2 cells and untreated LN-229 (which are tumour cells that incorporate more C-dots). Thus, we can assume that the autofluorescence of cells is no limitation to imaging applications with C-dots.

In summary, the observed anticancer activity of the as-synthesized spice-derived C-dots, in particular those from turmeric and black pepper, along with their preferential accumulation in glioblastoma cells, and excellent tolerability by non-cancerous cells, suggest great potential for a biomedical applicability of the spice C-dots as theranostic agents.

Further studies would be necessary to achieve a better understanding of the mechanism of action of these C-dots, and to clarify the pathways involved in the toxicity in both cell types. Black pepper C-dots showed the most noticeable effect on the LN-229 cells. Thus, in order to elucidate whether the black pepper C-dots effect might be attributed to the presence of molecules existing in the starting material, mass spectrometry measurements of black pepper C-dots were performed. It is known that piperine is the major compound of black pepper, and it is present in 5–10 wt % of the spice. Hence, identification of piperine in black pepper C-dots was accomplished using an ESI-QToF instrument Impact II by exact mass (*m*/*z* 286.1). Additionally, the sample was measured in multiple reaction monitoring mode (MRM, MS/MS) through selection of the piperine mass in the quadrupole and further analysis of the fragmentation pattern. The presence of piperine at trace level was confirmed by the fragmentation pattern when comparing with piperine standard at *m*/*z* 115.0, 201.1 and 286.1 (see Figure S10, Supporting Information File 1). Thus, the reduction of cancer cell viability produced by the assayed spice-derived C-dots could be attributed to the components of the spices employed for the synthesis. Even if present at low concentrations, the bioavailability of the active molecules from the spices could be increased when carried by the C-dots; for instance, their transport into cells could be increased or driven through different pathways that could lead to a different metabolization/degradation pathway.

## Conclusion

The results of this study demonstrate that C-dots showing similar physicochemical characteristics can be synthetized from diverse spices using a one-pot green hydrothermal method. Cinnamon, red chilli, turmeric and black pepper C-dots have shown high fluorescence quantum yields of 35.7, 26.8, 38.3 and 43.6%, and particle sizes measured by TEM of 3.37, 3.14, 4.32 and 3.55 nm, respectively. Additionally, the high values of negative zeta potential that all the spice-based C-dots presented ensure a great colloidal stability in biological media.

The four different spice-based C-dots have been systematically evaluated to study the in vitro toxicity to human cancer cells. The C-dots exhibit an interesting differential cytotoxicity in cancerous and non-cancerous human cells, which should be further explored. This differential cytotoxicity depends from the spice from which the C-dots were synthesized. An evident concentration-dependent reduction in cell viability was observed for LN-229 cells after 24 h of exposure to increasing concentrations of each C-dot type. In fact, results obtained showed that 2 mg/mL of cinnamon, red chilli, turmeric and black pepper C-dots yielded cancer cell growth inhibition efficiencies of 35, 50, 50 and 75%, respectively, whereas there was no significant growth inhibition to non-cancerous cells. Our preliminary results showed that this effect might be attributed to the presence of active molecules within the C-dot nanostructure, and more studies should be performed to understand the mechanism of action. Although it is of paramount importance to understand the complex nature of the C-dots, currently there is no agreement in the principles of the emission routes of the C-dots. A detailed investigation using non-conventional spectroscopic techniques would help to shed light on some missing information concerning C-dots. This information would allow for a better comprehension of how the C-dots interact with cancerous and non-cancerous cells with a selective cytotoxic effect, and more applications, including photodynamic therapy, could be devised.

Finally, results from the experiments allow us to unequivocally affirm that the C-dots synthesized here have a strong potential for bioanalytical and clinical applications. First, results obtained from fluorescence confocal microscopy studies have demonstrated that the C-dots from spices can be easily tracked when incorporated into cells, because their self-fluorescence is clearly different from the background of the medium. The excellent photoluminescence properties can be exploited for in vitro imaging applications, avoiding interferences from undesirable autofluorescence of cells significantly affecting conventional fluorophores. Moreover, depending on the source selected for the synthesis the C-dots exhibit different toxicological behaviour. In fact, tumour cell growth inhibition can be achieved by incubating the tumour cells with these C-dots. Such fundamental finding, not reported before, opens an exciting venue to explore future biomedical applications. In brief, the interesting anticancer activity of the spice-derived C-dots along with the bioimaging applicability and excellent tolerability in non-cancerous HK-2 cells, suggests a promising future potential as efficient theranostic agents with minimal side effects in non-cancerous cells.

## Experimental

### Chemicals

Cinnamon, red chilli, turmeric and black pepper powders were purchased from the local grocery store. 0.22 µm cellulose ester mixed Whatman filter paper, and 29.3 mm diameter dialysis tube (MWCO: 3.5 kDa) were obtained from Fisher Scientific (Portugal). Dulbecco’s modified Eagle’s medium (DMEM) with high glucose content and all other chemicals were purchased from Sigma-Aldrich (Spain). Ultrapure water (18.2 MΩ·cm at 25 °C, Millipore USA) was used throughout the experiments. Dulbecco’s modified Eagle’s medium with nutrient mixture F-12 (DMEM/F-12) and GlutaMAX-I™, trypsin 0.25%–EDTA, antibiotic mixture of penicillin/streptomycin (10,000 U·mL^−1^/10,000 μg·mL^−1^), fungizone (250 μg·mL^−1^), human transferrin (4 mg·mL^−1^) and phosphate buffered saline solution (1× PBS) were obtained from GIBCO Invitrogen (Barcelona, Spain). Fetal bovine serum (FBS) was obtained from HyClone GE Healthcare (United Kingdom). HK-2 (ATTC^®^ CRL-2190™) and LN-229 cells (ATTC^®^ CRL-22611™) were obtained from ATCC (LGC Standards S.L.U., Spain).

### Synthesis of C-dots

The C-dots were synthesized by a green one-pot hydrothermal method. Typically, 2.0 g of grounded spice (cinnamon, red chilli, turmeric and black pepper) were diluted in 10 mL of ultrapure water and sonicated for 30 min at 80 kHz, 25% ultrasonication power at 30 °C temperature (Elmasonic P 30 H ultrasonicator, Elma Schmidbauer GmbH, Germany). Afterwards the mixture was stirred for 15 min followed by a hydrothermal treatment at 200 °C for 12 h using Teflon coated autoclaves. Once finished, the resultant black carbonized solution was cooled down to room temperature. Subsequently, this solution was filtered through a 0.22 µm cellulose ester mixed Whatman filter paper in order to remove large particles. The brownish yellow filtrate solution was dialyzed in 1 L ultrapure water using a dialysis membrane with 3.5 kDa MWCO for 6 h, and the dialysis water was changed every 30 min. Finally, 1 mL of the purified C-dots was aliquoted and dried at 100 °C until a stable weight was obtained. Afterwards, based on the weight loss method, the concentration of C-dots was calculated.

### Characterization of C-dots

UV–visible spectroscopic measurements were performed on a Shimadzu UV-2550 UV–vis spectrophotometer (Shimadzu Corporation, Japan). Fluorescence spectra were measured using a Horiba Scientific Fluoromax-4 instrument (Horiba Scientific, USA), equipped with a xenon discharge lamp, 1 cm quartz cell at room temperature. For all the fluorescence measurements, excitation and emission slit widths were kept at 5 nm.

Dynamic light scattering (DLS) and zeta potential studies were performed on a Horiba nanoPartica SZ-100 instrument (Horiba Scientific, USA). For this purpose, 500 and 100 µL of the C-dots solution were placed into the disposable specific cuvettes. Both hydrodynamic radii and zeta potential measurements were performed at room temperature (*T* = 25 °C). Transmission electron microscopy (TEM) experiments were carried out with a JEOL-2100 transmission electron microscope (JEOL Ltd, Japan) working at 200 keV. The C-dots were placed onto formvar-carbon coated copper TEM grids with 400 mesh (Agar Scientific, UK) and dried under vacuum at room temperature before imaging.

X-ray diffraction measurements were taken in a X Pert PRO MRD diffractometer (PanAnalytical B.V, EA Almelo, The Netherlands) and Cu Kα radiation (λ = 0.15418 nm), and the samples were prepared on a Si substrate. Raman spectroscopy measurements were performed in Witec Alpha 300R confocal Raman Microscopy system with a 50× objective (WITec Wissenschaftliche Instrumente and Technologie GmbH, Germany) and the C-dots samples were prepared on a glass substrate. Fourier transform infrared (FTIR) spectra were recorded using a Perkin Elmer Spectra 100 FTIR Spectrometer (Perkin Elmer, USA).

Electrospray ionization coupled to quantitative time of flight mass spectrometry measurements were performed in an ESI-QTOF instrument Impact II (Bruker Daltonics, Bremen, Germany), working in positive detection mode. The mass range was recorded between 100 and 500 Da, using a capillary voltage of 4500 V. The nebulizer was working at 20 psi and the drying gas flow was kept at 4 L·min^−1^. The temperature was set at 200 °C, and the collision cell energy between 17.5 and 52.5 eV.

### Cell culture

HK-2 cells were grown in DMEM/F12 medium supplemented with 10% FBS, 100 U·mL^−1^ penicillin/100 μg·mL^−1^ streptomycin, 2.5 μg·mL^−1^ fungizone, and 5 μg·mL^−1^ human transferrin. LN-229 cells were maintained in DMEM high glucose, supplemented with 10% FBS and 100 U·mL^−1^ penicillin/100 μg·mL^−1^ streptomycin. Both cell lines (passages 5 to 12) were maintained in a humidified atmosphere with 5% CO_2_, at 37 °C.

### Cell viability test

Cells were seeded in 96-well plates at a density of 10,000 cells per well. On the following day, cells were incubated with growing concentrations of each C-dot solution (0.1 to 2.0 or 4.0 mg·mL^−1^) and cytotoxicity was evaluated 24 h after using PrestoBlue^®^ (PB, Invitrogen Corporation, San Diego, USA) viability reagent. This resazurin-based assay relies on the conversion, by viable cells, of the non-fluorescent compound resazurin to the highly fluorescent resofurin. Briefly, at the correspondent time-point, PB was added to each well further incubated for 1 h, at 37 °C at a 1:10 dilution. Fluorescence emission readings at 590 nm were performed using a Synergy HT microplate reader (BioTek^®^), using an excitation wavelength of 560 nm. To assess the level of interference of the C-dots on the technique used to measure cell viability, untreated cells were similarly incubated with PB to obtain the reduced compound. The obtained cell culture medium containing the reduced compound was separated from the cells and its fluorescence was measured at 560 nm before and after incubation with growing concentrations (0.1, 0.5, 1.0 and 2.0 mg·mL^−1^) of each type of C-dots. Results are means ± SD of the percentage of initial fluorescence.

### Cell imaging

Cells were seeded in 8-well glass bottom μ-slides (Ibidi, Martinsried, Germany) at a density of 30,000 cells per well. On the following day, cells were exposed to solutions of 1 mg·mL^−1^ of each C-dot type and incubated for 24 h, at 37 °C. After fixing the cells with a 4% paraformaldehyde solution (30 min, at 37 °C) and washing with PBS, cells were imaged using a Zeiss LSM780 laser scanning confocal microscope (Carl Zeiss Microimaging GmbH, Göttingen, Germany). Collected images were analysed using Zen 2010 software.

### Statistical analysis

All cell viability data (mean values ± SD of at least three independent experiments) were analysed using GraphPad Prism, version 6.0 (GraphPad Software, San Diego, CA). Differences in cell viability levels at each C-dots concentration, between citric acid-derived C-dots and each of the spice-derived C-dots, for each cell line, were estimated using regular two-way ANOVA followed by Dunnett’s multiple comparison post hoc test. The 0.05 level of probability was used as criterion of significance.

## Supporting Information

Supporting Information features emission spectra of cinnamon, red chilli and turmeric C-dots, as well as cell viability studies and ESI-QTOF spectra of black pepper C-dots and piperine standard.

File 1 Additional experimental data.
