# Supplementary material for: Green synthesis of fluorescent carbon dots from spices for in vitro imaging and tumour cell growth inhibition
Source: Beilstein J Nanotechnol. 2018 Feb 13;9:530–44. doi: 10.3762/bjnano.9.51 (PMC5827765; doi:10.3762/bjnano.9.51)
Supplement: File 1 — Additional experimental data. [file Beilstein_J_Nanotechnol-09-530-s001.pdf]

Supporting Information

for

Green synthesis of fluorescent carbon dots from spices for in vitro imaging and tumor cell growth inhibition

Nagamalai Vasimalai[§,1], Vânia Vilas-Boas[§,1,2], Juan Gallo[1], María de Fátima Cerqueira[3], Mario Menéndez-Miranda[4], José Manuel Costa-Fernández[4], Lorena Diéguez[1], Begoña Espiña[1] and María Teresa Fernández-Argüelles*[1]

Address: [1]Life Sciences Department, International Iberian Nanotechnology Laboratory (INL), Avenida Mestre José Veiga, 4715-330 Braga, Portugal; [2]UCIBIO-REQUIMTE, Laboratory of Toxicology, Biological Sciences Department, Faculty of Pharmacy, University of Porto, Rua de Jorge Viterbo Ferreira, 228, 4050–313 Porto, Portugal; [3]Center of Physics, University of Minho, 4710-057 Braga, Portugal and [4]Department of Physical and Analytical Chemistry, University of Oviedo Julian Clavería 8, 33006 Oviedo, Spain

Email: Maria Teresa Fernandez-Argüelles* - maria.fernandez-arguelles@inl.int

* Corresponding author

[§] Equal contributors

Additional experimental data

TABLE OF CONTENTS

1. Emission spectra of cinnamon, red chilli and turmeric C-dots
2. Cell viability studies
3. ESI-QToF/MS spectra of black pepper C-dots and piperine standard

1. Emission spectra of cinnamon, red chilli and turmeric C-dots

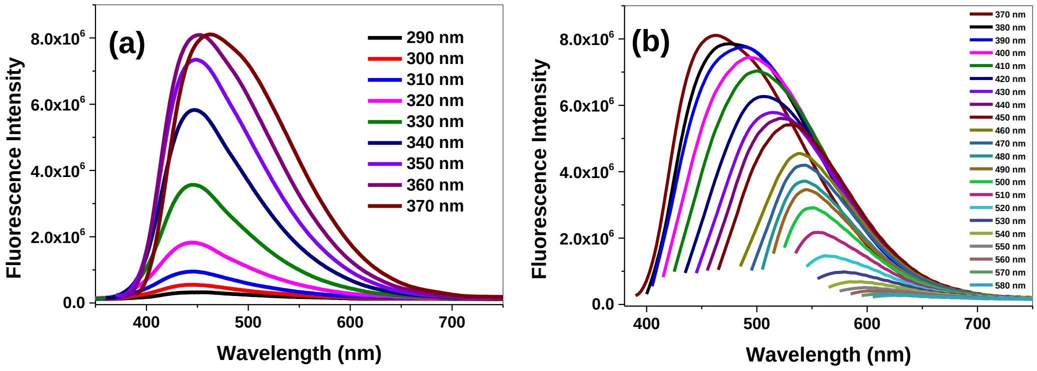

Figure S1: Emission spectra of cinnamon C-dots with different excitation (a) from 290 to 370 nm and (b) from 370 to 580 nm (λex/λem: 370/465 nm).

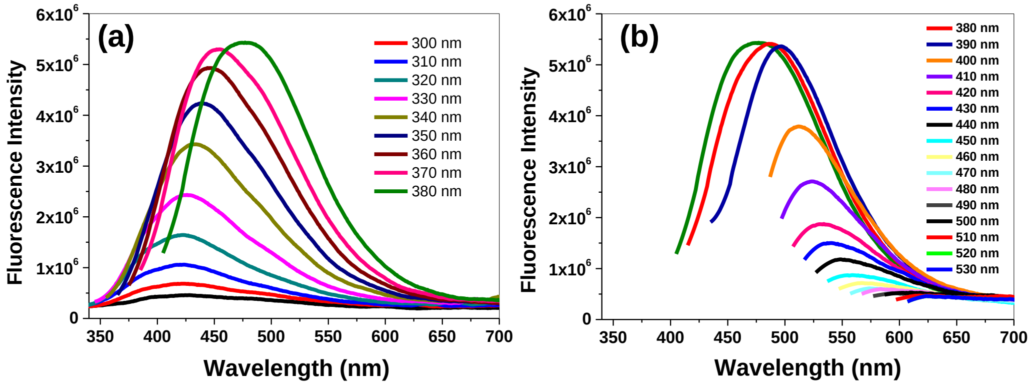

Figure S2: Emission spectra of chilli C-dots with different excitation (a) from 300 to 380 nm and (b) from 380 to 540 nm (λex/λem: 380/477 nm).

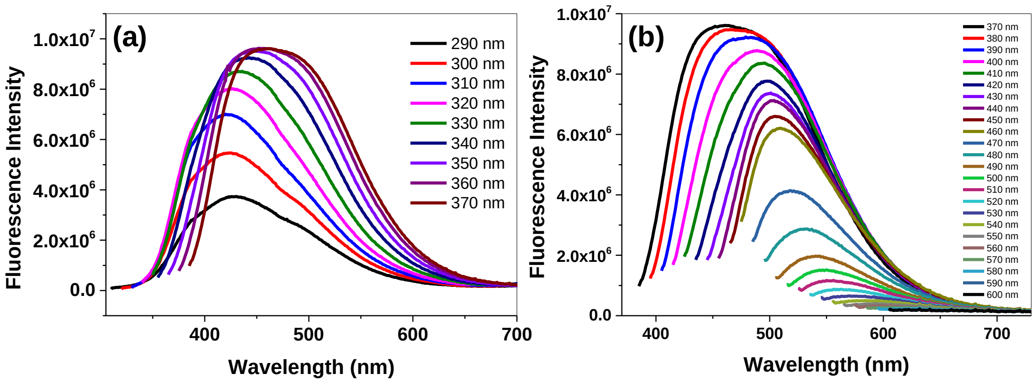

Figure S3: Emission spectra of turmeric C-dots with different excitation, (a) from 290 to 370 nm and (b) from 370 to 600 nm (λex/λem = 370/460 nm).

2. Cell viability studies

Untreated cells were incubated with PrestoBlue to obtain the reduced compound. The as obtained cell culture medium with reduced compound was separated from the cells and used to assess the level of interference of the C-dots on the technique used to measure cell viability. Fluorescence at 560 nm was measured before and after incubation with growing concentrations (0.1, 0.5, 1.0 and 2.0 mg/mL C-dots) of each type of C-dots. Results are means+SD of the % of initial fluorescence (cell culture medium only). Results obtained are displayed in Figure S4.

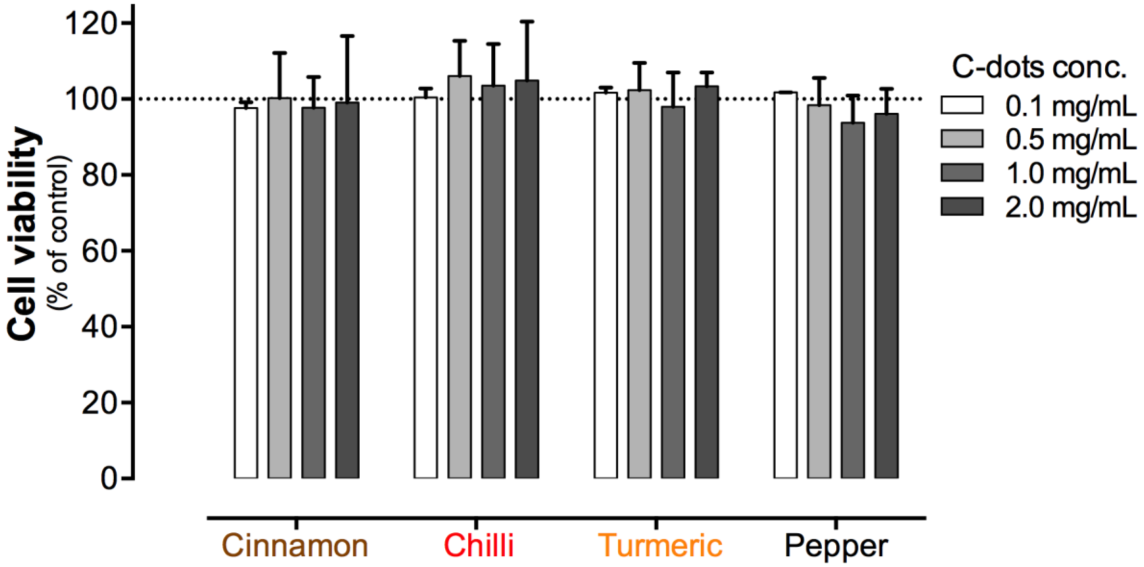

Figure S4: Excluding the interference of C-dots on the PrestoBlue assay.

Figures S5 to S9 show the results from cell viability studies performed 24h after incubation with each of the tested C-dots and compare the effects between the cell lines for the same concentration of the respective C-dots. Represented results are mean ± SD from at least three independent experiments and the statistical significance of differences was estimated using regular two-way ANOVA followed by Sidak's multiple comparisons post hoc test. *$p < 0.05$; ***$p < 0.001$ and ****$p < 0.0001$ vs the effect of the same concentration in the other cell line.

For all the tested food-derived C-dots a significant anti-cancer effect was observed when compared to the effects in normal HK-2 cells.

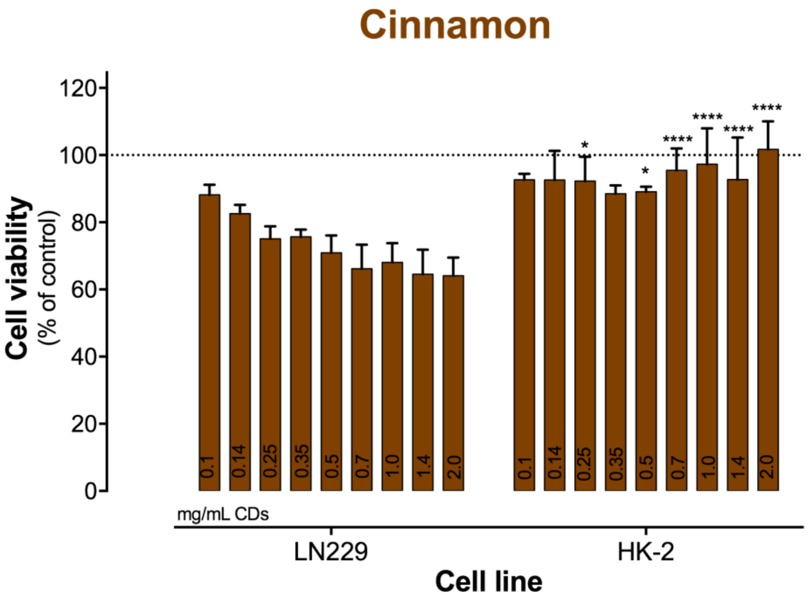

Figure S5: Cell viability evaluation after a 24 h incubation with growing concentrations of cinnamon C-dots. LN229 cell viability was overall significantly different from HK-2's, in particular for the highest tested concentration, which induced around 35 % decrease in cell viability of LN-229 cells while leaving the normal HK-2 cells undamaged.

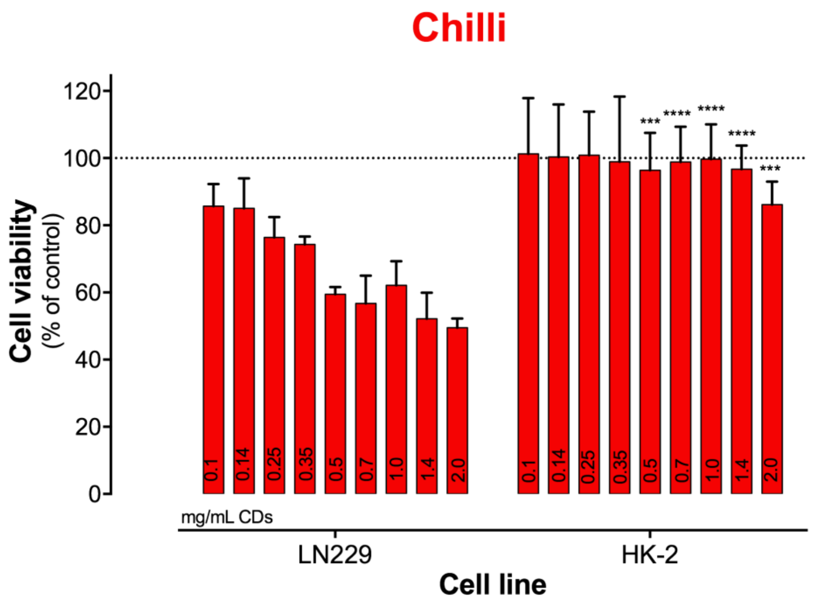

Figure S6: Cell viability evaluation after a 24 h incubation with growing concentrations of red chilli C-dots. The effects of the highest tested concentrations yielded around 50% decrease in LN-229 cell viability while the HK-2 cells were not affected by the presence of the chilli C-dots.

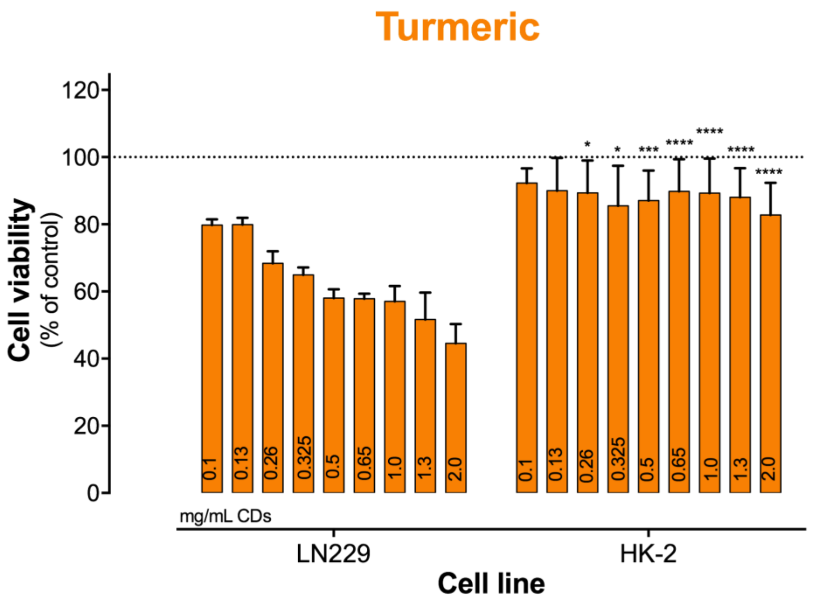

Figure S7: Cell viability evaluation after a 24 h incubation with growing concentrations of turmeric C-dots. As with chilli C-dots, turmeric C-dots induce a 50% reduction of LN-229 cell viability, an effect that is significantly different from the one observed with the normal HK-2 cells.

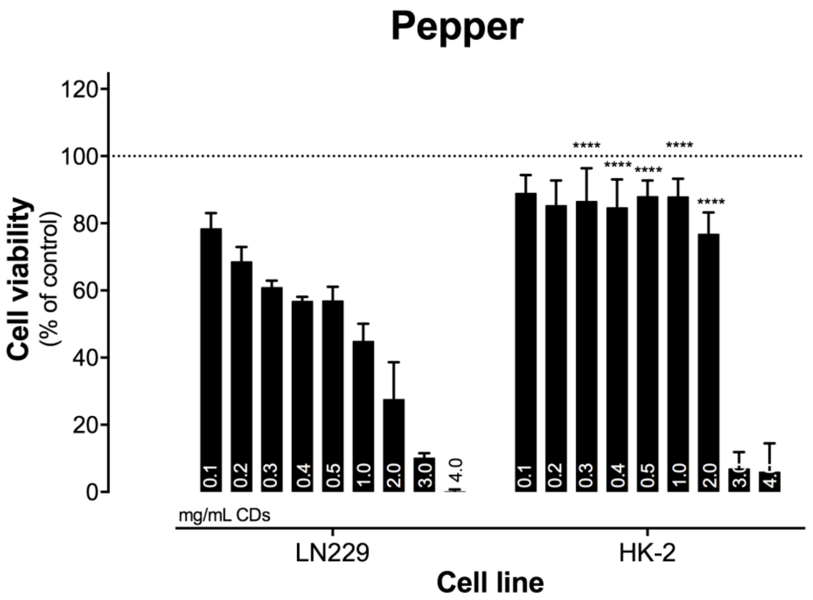

Figure S8: Cell viability evaluation after a 24 h incubation with growing concentrations of black pepper C-dots. These were the most effective anti-cancer C-dots inducing an approximately 75% reduction in the human glioblastoma cell line (LN-229) at 2 mg·mL^{-1}. A significantly different effect was observed for the HK-2 cells as 80% of the cells remain viable in the same conditions.

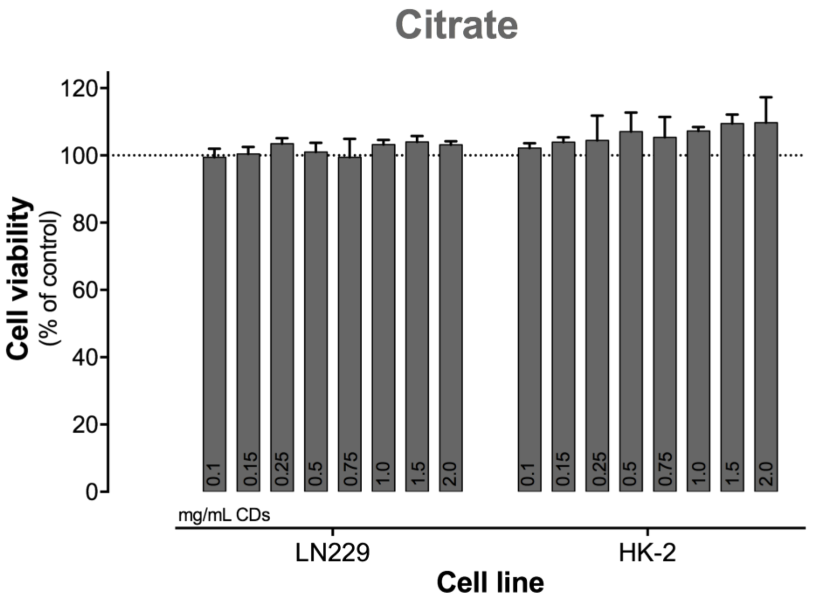

Figure S9: Cell viability evaluation after a 24 h incubation with growing concentrations of citrate C-dots.

3. ESI-QToF spectra of black pepper C-dots and piperine standard

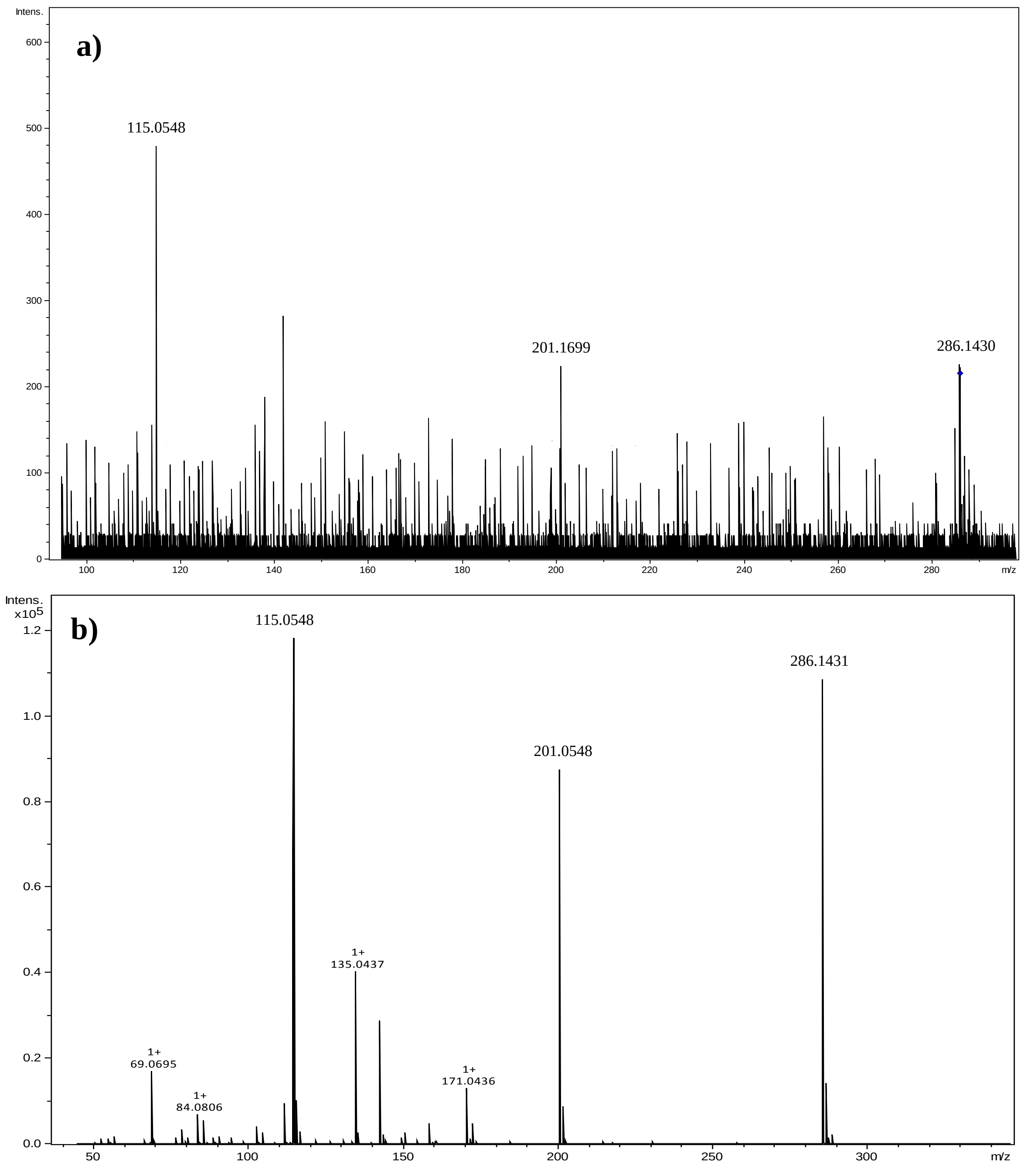

Figure S10: ESI-Q-TOF MS/MS Spectrum Piperine: a) pepper C-dots; b) piperine Standard (400 ppb).
